# European society of urogenital radiology (ESUR) guidelines: MR imaging of pelvic endometriosis

**DOI:** 10.1007/s00330-016-4673-z

**Published:** 2016-12-05

**Authors:** M. Bazot, N. Bharwani, C. Huchon, K. Kinkel, T. M. Cunha, A. Guerra, L. Manganaro, L. Buñesch, A. Kido, K. Togashi, I. Thomassin-Naggara, A. G. Rockall

**Affiliations:** 1 Department of Radiology, Tenon Hospital, 58 Avenue Gambetta, Paris, 75020 France; 2 Department of Radiology, St Mary’s Hospital, Imperial College Healthcare NHS Trust, 3rd Floor Queen Elizabeth the Queen Mother Building, Praed Street, London, W2 1NY UK; 3 Department of Obtetrics and Gynaecology, CHI Poissy Saint-Germain en Laye,Versailles University France, 10 rue du champ Gaillard, 78300 Poissy, France; 4 Institut de radiologie, Clinique des Grangettes, 7, chemin des Grangettes, CH 1224 Chêne-Bougeries, Switzerland; 5 Serviço de Radiologia, Instituto Português de Oncologia de Lisboa Francisco Gentil, Rua Prof. Lima Basto, 1099-023 Lisboa, Portugal; 6 Department of Radiology, Hospital da Luz, Lisbon, Portugal; 7 Department of Radiological Sciences, Sapienza University of Rome, Vle. Regina Elena 324, 00162 Rome, Italy; 8 Department of Radiology (Urogenital Section), Hospital Clínic Barcelona, Villarroel, 170, Barcelona, 08036 Spain; 9 Department of Diagnostic Radiology, Kyoto University Hospital, 54 Shogoin-Kawahara-cho, Sakyo-ku, Kyoto, 606-8507 Japan; 10 Department of Radiology, The Royal Marsden Hospital, Fulham Road, London, SW3 6JJ UK

**Keywords:** Endometriosis, Guidelines, Magnetic resonance imaging (MRI), Protocols, Evidence-based Medicine/standards

## Abstract

**Abstract:**

Endometriosis is a common gynaecological condition of unknown aetiology that primarily affects women of reproductive age. The accepted first-line imaging modality is pelvic ultrasound. However, magnetic resonance imaging (MRI) is increasingly performed as an additional investigation in complex cases and for surgical planning. There is currently no international consensus regarding patient preparation, MRI protocols or reporting criteria. Our aim was to develop clinical guidelines for MRI evaluation of pelvic endometriosis based on literature evidence and consensus expert opinion. This work was performed by a group of radiologists from the European Society of Urogenital Radiology (ESUR), experts in gynaecological imaging and a gynaecologist expert in methodology. The group discussed indications for MRI, technical requirements, patient preparation, MRI protocols and criteria for the diagnosis of pelvic endometriosis on MRI. The expert panel proposed a final recommendation for each criterion using Oxford Centre for Evidence Based Medicine (OCEBM) 2011 levels of evidence.

***Key Points*:**

• *This report provides guidelines for MRI in endometriosis*.

• *Minimal and optimal MRI acquisition protocols are provided*.

• *Recommendations are proposed for patient preparation*, *best MRI sequences and reporting criteria*.

**Electronic supplementary material:**

The online version of this article (doi:10.1007/s00330-016-4673-z) contains supplementary material, which is available to authorized users.

## Introduction

Endometriosis is a common gynaecological condition that is defined as functional ectopic endometrial glands and stroma outside the uterus. This disease affects women of reproductive age, with a prevalence of approximately 10% [1]. Patients can be asymptomatic or present with chronic pelvic pain and/or infertility. The combination of laparoscopy and histological verification of endometrial glands and/or stroma is considered to be the gold standard for diagnosis of the disease [2].

Ultrasonography (US) is the first-line imaging modality for the assessment of pelvic endometriosis but has limitations with respect to field-of-view and operator dependence [3–8]. Magnetic resonance imaging (MRI) is usually performed as an additional examination in complex cases or prior to surgery and is highly accurate in the evaluation of endometriosis [9–18]. However, there is no international consensus on patient preparation, best MRI sequences or reporting criteria.

The aim of this work was to develop guidelines for optimal MRI protocols and image interpretation in endometriosis based on a detailed literature review and consensus expert opinion from the Female Pelvic Imaging working group of the European Society of Urogenital Radiology (FPI-ESUR).

## Methods

MRI protocols for the diagnosis of pelvic endometriosis were collected from one non-European and seven European institutions. Inclusion criteria to participate in the guideline development process were: to be a member of the FPI-ESUR working group, and to perform regular MRI examinations and/or publications related to endometriosis. All but one investigators included in this guidelines process were experts in gynaecological imaging, the last being a gynaecologist expert in methodology.

A questionnaire was established containing the following information: patient preparation, magnet field strength, type of coil, type of MRI sequences, use of intravenous contrast injection, and vaginal and/or rectal opacification.

Published literature was reviewed through a Medline literature search of abstracts in the English language of studies in human subjects, including the following keywords: ‘endometriosis’ and ‘MR imaging’ up to June 2015. Articles that did not include technical details matching the information requested in the questionnaire were excluded. The details were entered into an Excel spread sheet and the results discussed and divided into topics with agreement and disagreement. Topics with disagreement were compared to the literature. Experts in favour of one technical option were asked to support their views using data from the literature. The expert panel proposed a final recommendation for each criterion using Oxford Centre for Evidence Based Medicine (OCEBM) 2011 levels of evidence

The summary of valid scientific data for each question analysed by the experts included a level of evidence (LE) based on the quality of available data and defined according to the rating scheme developed by the French National Authority for Health:

- LE1: high-power randomized comparative trials or meta-analyses of randomized comparative trials;
- LE2: low-power randomized trials, well-conducted non-randomized comparative studies and cohort studies;
- LE3: non-consecutive studies or studies without consistently applied reference standards;
- LE4: non-randomized comparative studies with substantial bias, retrospective studies, cross-sectional studies and case series.

The practice guidelines were summarised from the responses provided by the experts, and grades were attributed as follows: Grade A: established scientific evidence (LE1); Grade B: scientific presumption (LE2); Grade C: based on a low level of evidence (LE3 or LE4). Recommendations based on professional consensus were reduced to a minimum (Good Practice Point: GPP) and were used in the queries when literature was lacking in evidence.

## Results

### Indications for MRI in endometriosis

No data exist in the literature about the indications for MR imaging for pelvic endometriosis: evaluation of pelvic pain, infertility or indeterminate adnexal mass. In accordance with the analysis performed in our different ESUR centres, more than 90% of MRI examinations are performed for staging deep pelvic endometriosis that is the main indication. Hence, an indeterminate adnexal mass represents an ancillary indication of MR imaging.

No consensus exists in the literature regarding the use of MRI in comparison to US. In practice, radiological papers tend to favour MRI whereas gynaecological publications underline the value of US [19, 20]. This assessment is in line with recent published meta-analyses [21–24]. The first confirmed that transvaginal sonography (TVS) should remain the first-line method in the evaluation of patients with suspicion of deep pelvic endometriosis (DPE) [21]. Two further meta-analyses demonstrated that the overall diagnostic performance of TVS for detecting DPE is fair but a high specificity is present for all locations [22, 23]. Based on the results of these meta-analyses, further investigations, especially MRI, are recommended in a symptomatic patient in the presence of negative US findings (LE1) [22, 23]. Finally, an additional meta-analysis suggested that MRI is a useful preoperative test for predicting the diagnosis of multiple sites of DPE (LE1) [24].

In patients with equivocal US, MRI is recommended as a second-line technique in the preoperative workup of DPE (grade A).

### Technical requirements

Technical requirements for each centre are presented in Table 1. A summary of literature review is provided for each specific criterion.

**Table 1:** Technical requirements

	Paris	London	Geneva	Lisbon	Lisbon	Roma	Barcelona	Kyoto
Device (Tesla)	1.5	1.5/3.0	3.0	1.5	1.5/3.0	3.0	1.5/3.0	1.5/3.0
Phased-array	Yes	Yes	Yes	Yes	Yes	Yes	Yes	Yes
Endocavitary probe	No	No	No	No	No	No	No	No
Timing of MRI	No	No	> Day 8	No	No	No	No	No
Fasting	3h	No	6h	6h	4h	6h	4h	4h
Special diet	No	No	No	No	No	No	No	No
Bowel enema	Yes	No	Yes	No	Yes	Yes	No	No
Bladder emptying	2h	No	No	2h	1h	1h	No	No
IV catheter	No (Option)	Yes	Yes	Yes	Yes	Yes	Yes	No
Anti-peristaltic agent	SC	IV	IM	IV	IV	IV	IV	SC
Belt strapping	Yes	No	Yes	Yes	No	No	No	Yes
Vaginal opacification	No	No	Yes^*^	Yes^*^	Yes^*^	No	No	No
Rectal opacification	No	Yes	Yes^*^	Yes^*^	Yes^*^	Yes^*^	No	No
Supine position	Yes	Yes	Yes	Yes	Yes	Yes	Yes	Yes
Prone position	Yes^**^	No	No	No	No	No	No	No

*IM* intramuscular, *IV* intravenous, *SC* subcutaneous

^*^ If doubt or symptoms present (i.e. dyspareunia, dyschezia)

^**^If claustrophobic

### 1.5 Tesla versus 3.0 Tesla systems

The majority of published studies use a 1.5T magnet. Only four publications used 3.0T but suggested promising results [11, 16, 25, 26]. At 3.0T, improved signal-to-noise ratio results in the acquisition of high-spatial resolution images and accurate depiction of all locations of DPE [11, 16, 25]. However, there is increased image heterogeneity at 3.0T when compared to 1.5T, which can have a negative effect on the fat-saturation techniques routinely utilised in the evaluation of endometriosis [16, 27]. The application of the Dixon technique may overcome this and achieve stronger fat-suppression [27].

**Fig. 1:**
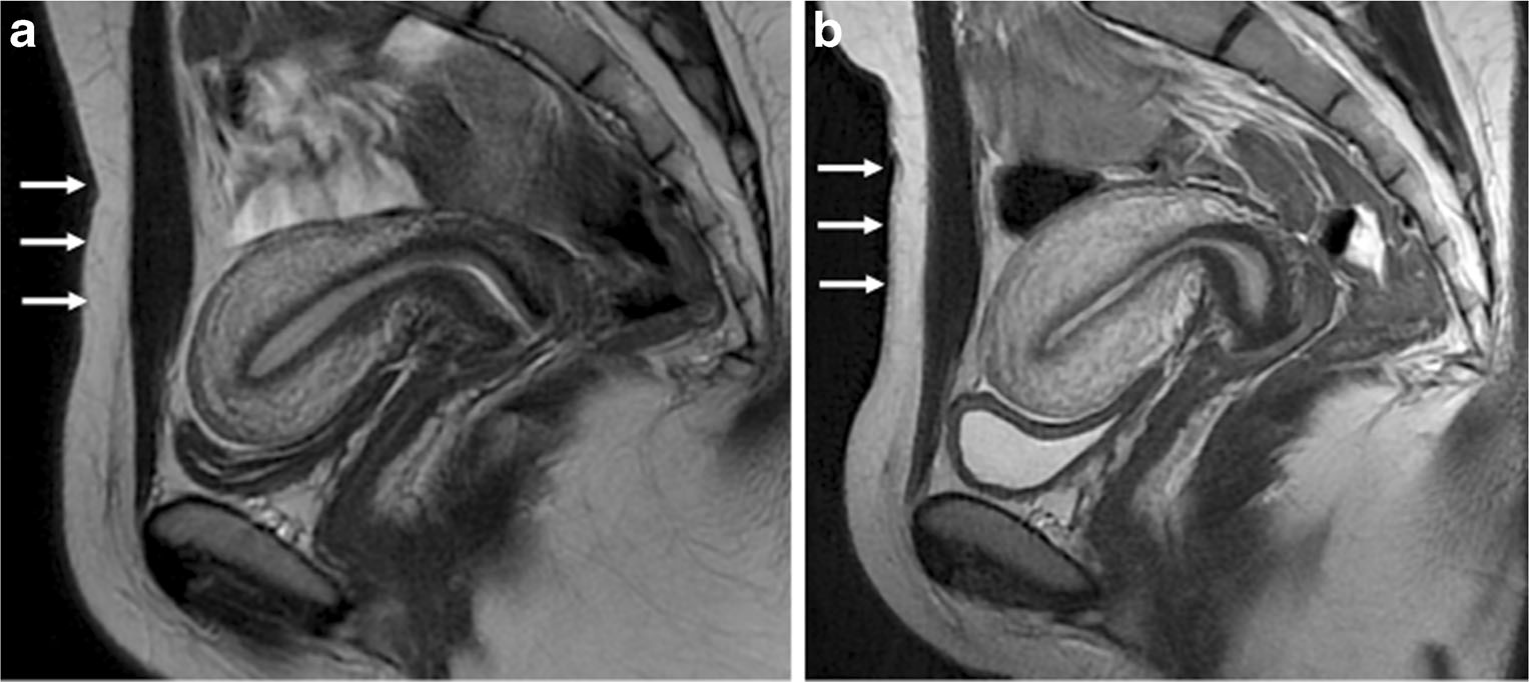
Sagittal 2D T2-weighted MR images in the same patient performed at (**a**) 1.5 Tesla and (**b**) 3.0 Tesla provide similar good imaging quality for the evaluation of pelvic anatomy, especially uterine zonal anatomy. Note the quality of abdominal strapping on both 1.5 and 3.0 T examinations (arrows)

Both 1.5T and 3.0T seem valuable for the evaluation of DPE; however, studies comparing the systems are lacking. Therefore, no recommendation can be made for the use of a specific device and further work is necessary to perform this comparison (Fig. 1).

### Array type

In line with different publications, pelvic phased-array coils provide a higher SNR than is possible with a body coil (LE3) [28, 29]. In addition, three studies have reported additional value of endocavitary coils in conjunction with pelvic phased-array [9, 30, 31]. Drawbacks in terms of cost and acceptability limit its potential use in the evaluation of DPE.

Pelvic phased array coils are recommended in the evaluation of DPE at both 1.5T and 3.0T (grade C).

### Timing of MRI examination

**Fig. 2:**
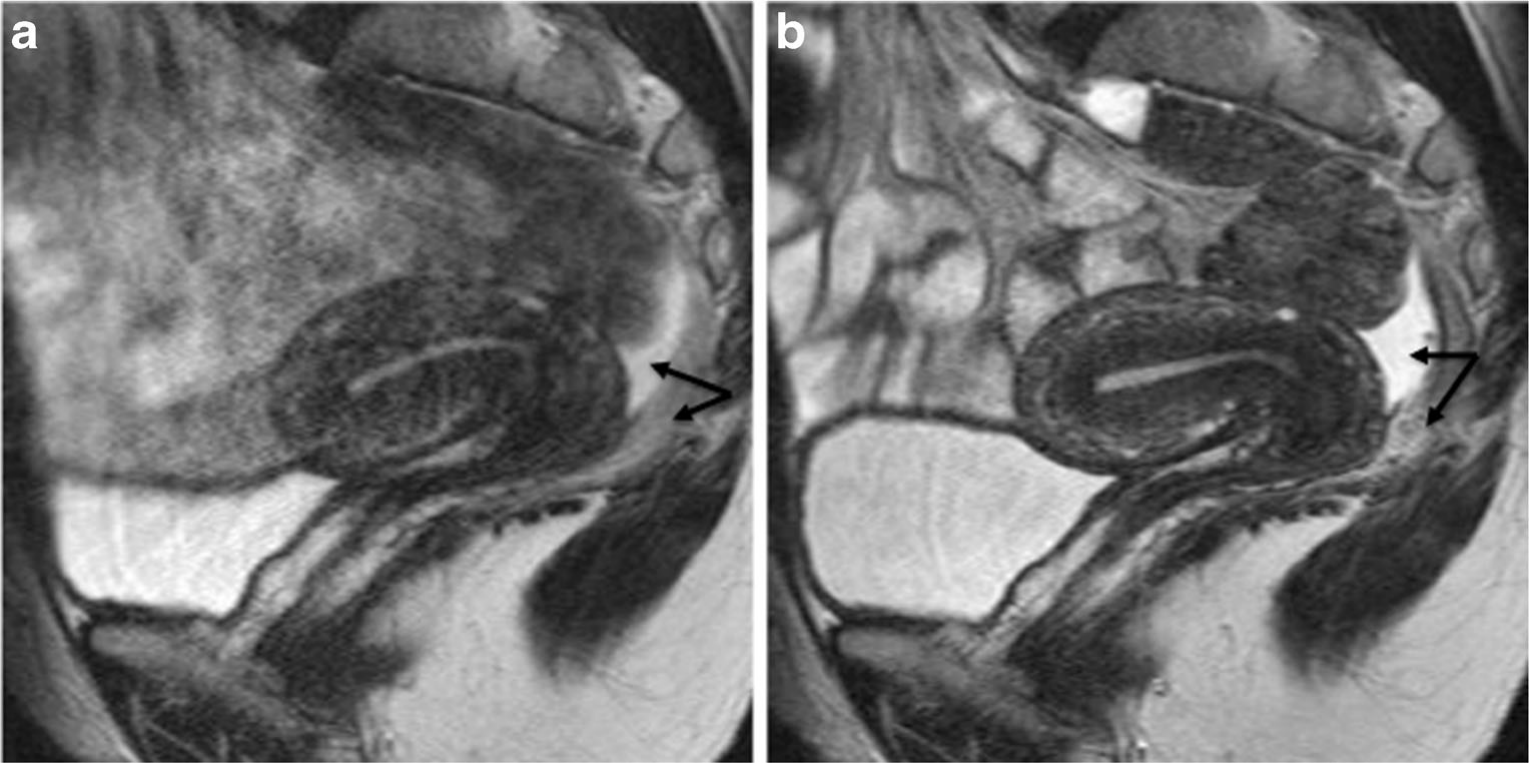
Sagittal 2D T2-weighted MR images performed at 1.5 Tesla showing the benefits of anti-peristaltic agents on image quality. Imaging performed in the same patient before (**a**) and after (**b**) administration of glucagon demonstrating a dramatic improvement in image quality. Note the presence of pelvic fluid in the pouch of Douglas underlining a clear demarcation between peritoneal and posterior subperitoneal compartments (double arrow) (reprinted with permission - Bazot M. Ed. Lavoisier-Paris 2016)

Several studies reported discrepant results regarding timing of MRI evaluation. Fiaschetti et al. examined patients between days 8 and 12 of the menstrual cycle, due to the possibility of spontaneous T1W -signal intensity of blood prior to day 8 of the menstrual cycle [32]. Bazot et al. suggested that the presence of pelvic free fluid (e.g. menstruation, post-ovulatory phase) is a useful aid to image interpretation (Fig. 2) [33, 34]. Tamai et al. reported that during menstruation the uterus can demonstrate marked pseudo-thickening of the junctional zone, suggesting an inappropriate diagnosis of adenomyosis [35]. They went on to suggest that MRI should be avoided in the menstrual phase [35]. In addition, these authors reported that the evaluation of uterine peristalsis is optimal during the peri-ovulatory phase [36]. Solak et al. reported no significant difference in size of lesions in the early days of menstruation compared to the mid-menstrual period for abdominal wall endometriosis [37]. Finally, Botterill et al. did not observe a significant difference in disease extent between menstruating and non-menstruating scans [38].

No recommendation can be proposed for timing of MRI in relation to the menstrual cycle in the evaluation of DPE.

### Patient preparation

There was no consensus regarding patient preparation before MRI. The committee felt that the protocol should be tailored to the main indication for pelvic MRI (diagnosis/staging of DPE, indeterminate adnexal mass).

### Fasting

When fasting prior to the MRI study was mentioned, the length of fast was variable at 3, 4 or 6 h (LE2) [16, 17, 19, 20, 32, 33, 39]. However, the majority of studies did not mention this pre-imaging preparation.

Fasting is recommended in the evaluation of DPE (grade B).

### Bowel preparation

Most studies did not mention the use of bowel preparation prior to pelvic MRI. Where authors advocated the use of bowel preparation, the type of preparation varied. The most commonly utilised method was bowel enema with either rectal suppository pills (e.g. bisacodyl) or water [39, 40]. In addition, there was variable use of dietary preparation, ranging from nothing to low-residue diet 3 days prior to MRI accompanied by enema, magnesium sulphate and fluid re-hydration the day before the study [41].

**Fig. 3:**
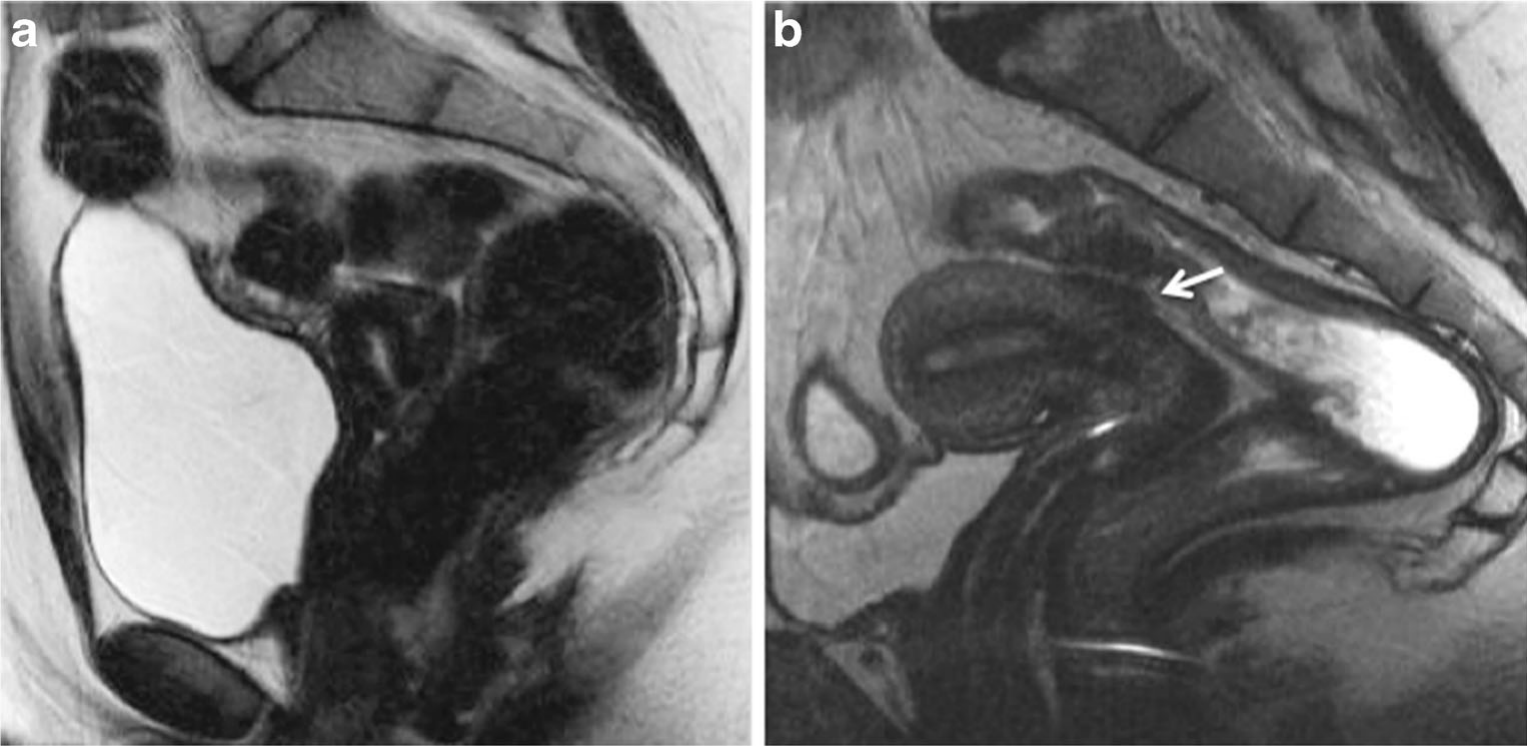
Sagittal 2D T2-weighted MR images performed at 1.5 Tesla showing the benefits of patient preparation on image quality. (**a**) Imaging performed with a full urinary bladder and without bowel preparation is sub-optimal for interpretation and disease may be overlooked. (**b**) MR imaging performed in a different patient following bowel preparation with Normacol and 2 h after emptying her urinary bladder. Note the superior image quality in (**b**) and the large endometriotic lesion on the anterior rectosigmoid colon (arrows)

Bowel preparation is advocated as ‘best practice’ for the detection of DPE (GPP) (Fig. 3).

### Bladder emptying

No studies have been published in the medical literature addressing the importance of bladder distension for detection of anterior DPE. When bladder distension is discussed, authors describe a moderately filled or full bladder in order to correct the angle of uterine anteversion and thereby improve visualisation of the region, allowing detection of small nodules located anterior to or in the vesicouterine pouch and to displace the bowel superiorly reducing artefact caused by bowel motion (LE4) [12, 16, 39, 42–46]. Excessive bladder distension is not recommended as associated detrusor contractions may cause artefact and can complicate the identification of small parietal nodules (LE4) (Fig. 3) [42, 44, 46]. To achieve the appropriate distension, authors mainly ask their patients not to empty their bladder for 1 h prior to the examination [16, 39].

A moderately full bladder is recommended in the evaluation of DPE (grade C).

### Patient position

All centres performed MRI with a patient in the supine position.

A recent systematic review specifically looked at possible interventions aimed to reduce anxiety, distress and the need for sedation in adults undergoing MRI exams, and confirmed evidence for the benefit of prone scanning in reducing claustrophobia (LE2) [47].

The supine position is recommended in the evaluation of pelvic endometriosis (GPP). The prone position is an ‘option’ in claustrophobia (grade B).

### Abdominal strapping

A few papers recommend the use of a broad abdominal belt in MRI examinations for the evaluation of endometriosis (Fig. 1) [34, 48, 49]. The purpose is to reduce artefact caused by respiratory movement and it has been recommended to apply the belt at the end of expiration (LE3) [50, 51].

Abdominal strapping is recommended in the evaluation of pelvic endometriosis (grade C).

### Anti-peristaltic agent

The use of an anti-peristaltic agent (e.g. glucagon, butyl-scopolamine), unless contraindicated (e.g. diabetes or phaeochromocytoma), is the most efficient way to limit bowel motion artefact (Fig. 2) (LE4) [52]. Recently, Gutzeit et al. suggested that intravenous spasmolysis is more reliable than intramuscular administration, and glucagon is better than butyl-scopolamine [52].

An anti-peristaltic agent is recommended in the evaluation of DPE (grade C).

### Vaginal opacification

**Fig. 4:**
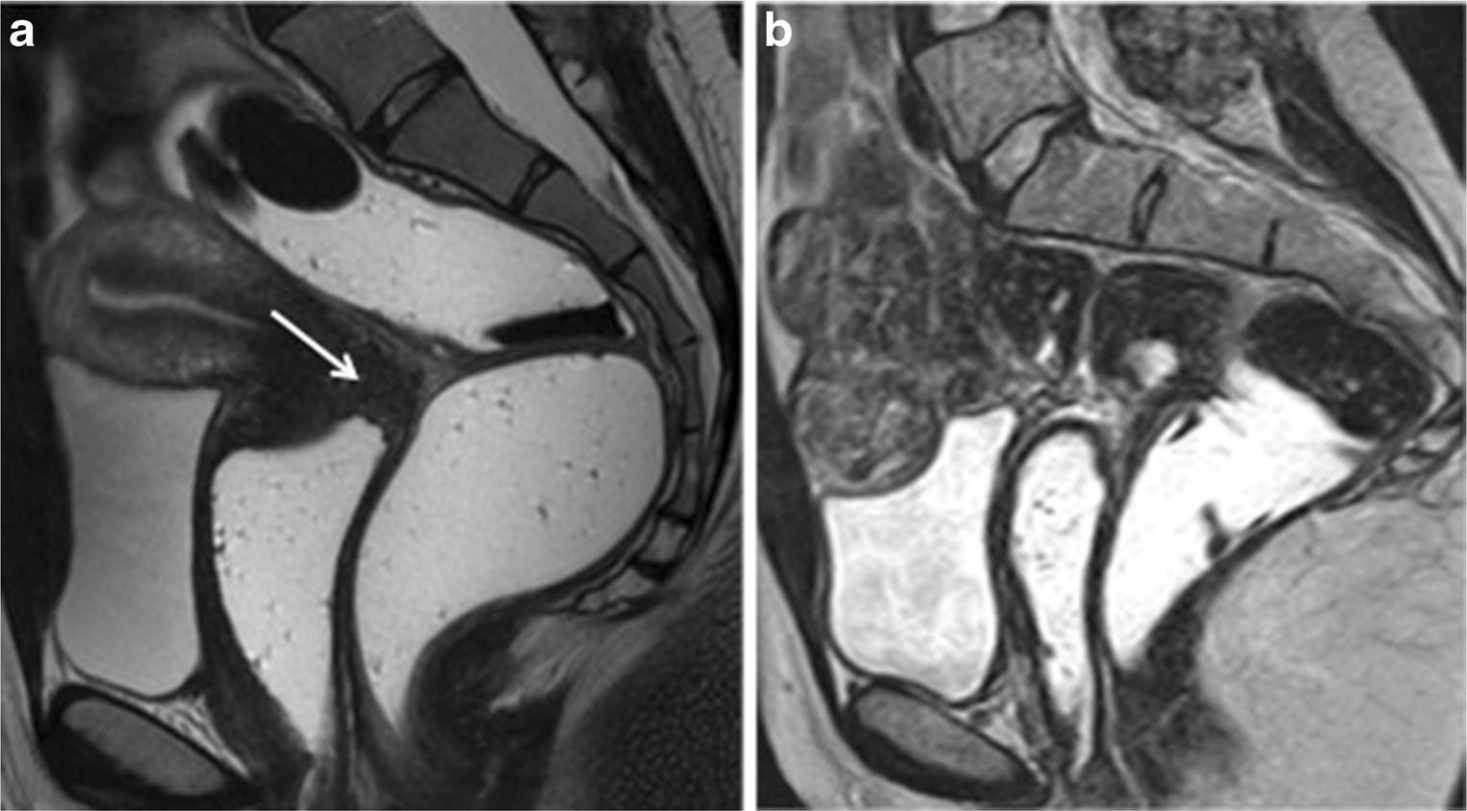
Sagittal 2D T2-weighted MR images performed in two different patients at 1.5 Tesla following vaginal and rectal opacification with sonographic gel and with (**a**) or without (**b**) bowel preparation. Vaginal distension demonstrates thickening of the posterior vaginal fornix (white arrow) without involvement of the pouch of Douglas or rectum posteriorly that is clearly analysable (**a**). Vaginal and rectal opacification without bowel preparation cannot permit an accurate analysis of potential deep posterior endometriosis, especially potential rectal endometriosis (**b**)

Four studies provided discrepant results on the value of vaginal opacification with gel in the diagnosis of posterior DPE (Fig. 4) (LE4) [14, 32, 45, 53]. The first reported an improvement in sensitivity between pre- and post-contrast MRI in the diagnosis of DPE; however, this improvement was only significant for junior radiologists [45]. The second did not find any significant difference in the diagnosis of vaginal or rectal endometriosis with or without vaginal opacification, whatever the level of expertise of readers (LE4) [14]. The third reported better evaluation for the detection of vaginal and uterosacral endometriosis but not for pouch of Douglas or rectovaginal septum disease (LE4) [32]. Finally, the most recent study reported a significant improvement in the diagnosis of pouch of Douglas obliteration in the presence of vaginal opacification (LE4) [53].

Vaginal opacification with sonographic gel is considered as an ‘option’ in the evaluation of DPE (GPP).

### Rectal opacification

No consensus exists in the literature on the value of rectal opacification in the diagnosis of DPE (Fig. 4). In practice, two different types of contrast medium are used (sonographic gel or water) [11, 14, 45, 53]. Discrepant results are available with some authors claiming that rectal opacification provides a better evaluation of pouch of Douglas and rectosigmoid colon endometriosis [32, 41, 43, 53], while several other studies argued that this technique was useless in the evaluation of posterior DPE locations [10, 11, 14]. In this setting, different arguments against systematic rectal opacification are suggested including time, patient discomfort, movement artefact and rectosigmoid colon spasm [10].

Rectal opacification is suggested as an ‘option’ in the evaluation of pelvic endometriosis (GPP).

### MRI protocol

#### MRI sequences (Table 2)

**Table 2:** MRI sequences

MRI sequence	Paris	London	Geneva	Lisbon	Lisbon	Roma	Barcelona	Kyoto
2DT2W sagittal	Yes	Yes	Yes	Yes	Yes	Yes	Yes	Yes
2DT2W axial	LP^*^	P^*^	LP^*^	LP^*^	LP^*^	P^*^	P^*^	P^*^
2DT2W coronal	No	No	Yes	No	Yes	Yes	Yes	No
2DT2W oblique	Yes	Yes	Yes	Yes	Yes	Yes	No	No
3DT2W	Yes	No	No	No	No	Yes	No	No
T2^*^	No	No	No	No	No	No	No	No
SSFSE/Haste	Yes	No	Yes	No	No	No	No	Yes
2D/3D T1W	3D	2D	2D	2D	3D	3D	2D	2D
T1W no FS^§^	Yes	Yes	Yes	Yes	Yes	Yes	Yes	Yes
T1W with FS^§^	Yes	Yes	Yes	Yes	Yes	Yes	Yes	Yes
Gadolinium	±	±	±	±	±	±	±	±
Peristalsis	±	No	No	No	No	No	No	Yes
DWI	No	No	No	No	No	Yes	No	Yes

*T1W* T1-weighted, *T2W* T2-weighted, *2D* two-dimensional, *3D* three-dimensional

LP^*^: from renal hila to pubic bone

P^*^: from iliac crests to pubic bone

T2^*^: susceptibility-weighted MR sequence

FS^§^: fat-saturation technique

There is significant variability in the literature regarding the MRI protocols used [11, 16–18, 31–33, 44, 45, 54–56].

#### T2-weighted MRI

T2W MR sequences without fat-suppression technique are the best sequences for detecting pelvic endometriosis (LE2) [33]. Most MRI studies are performed using at least two orthogonal T2W planes [11, 16–18, 31–33, 44, 45, 54–56]. Further studies are required to clarify the field-of-view used for the axial acquisition and which additional T2W MR plane should be used. Axial 2D-T2W MRI from renal hila to pubic bone, allowing a systematic visualisation of kidneys and potential analysis of the right iliac fossa (i.e. caecum, appendix, small bowel) should be recommended [33]. The use of thin section-oblique 2D-T2W imaging improves the success of conventional MRI (sagittal and axial) for assessment of uterosacral and parametrial endometriosis (LE3) [13, 15]. In addition, several authors have recently reported the potential value of 3D-T2W imaging in the evaluation of DPE [16, 54]. In contrast, no studies have demonstrated the value of coronal 2D-T2W MRI sequence in the evaluation of pelvic endometriosis.

Three 2D-T2W MRI sequences (sagittal, axial, oblique) are recommended in the evaluation of DPE (grade B).

The addition of 3D-T2W MRI sequence is proposed as an ‘option’ (grade C).

#### T1-weighted MRI

Several studies have underlined that T1W MRI is the gold standard for the diagnosis of endometriotic cysts (LE2) [57, 58]. The 2D or 3D Dixon technique providing four simultaneous different T1W contrasts during the same acquisition and a stronger fat suppression in the female pelvis when compared to a 3D-FSPGR sequence should progressively become the reference technique (LE4) [58]. There has been no comparative study between conventional fat saturated T1W sequences and the Dixon technique in the identification of endometrial implant. The reduced spatial resolution of currently available Dixon techniques might prevent the identification of small peritoneal implants compared to conventional fat-saturated T1W sequences. This hypothesis requires further research.

Data are lacking for the evaluation of DPE using T1W MRI.

Preliminary papers have suggested fat-suppressed T1W MRI to be of value in the diagnosis of peritoneal endometriosis, but this finding must be confirmed [59, 60].

T1W MRI sequences without and with fat suppression are recommended in the evaluation of adnexal endometriosis (grade B)

The ‘Dixon technique’ may be used as an alternative to standard T1W sequence (grade C).

#### Intravenous contrast-enhanced MRI

Few data are available regarding the value of gadolinium in the evaluation of endometriosis. A clear distinction must be made regarding the indication for MRI (diagnosis/staging of endometriosis/characterisation of US-indeterminate adnexal mass).

**Fig. 5:**
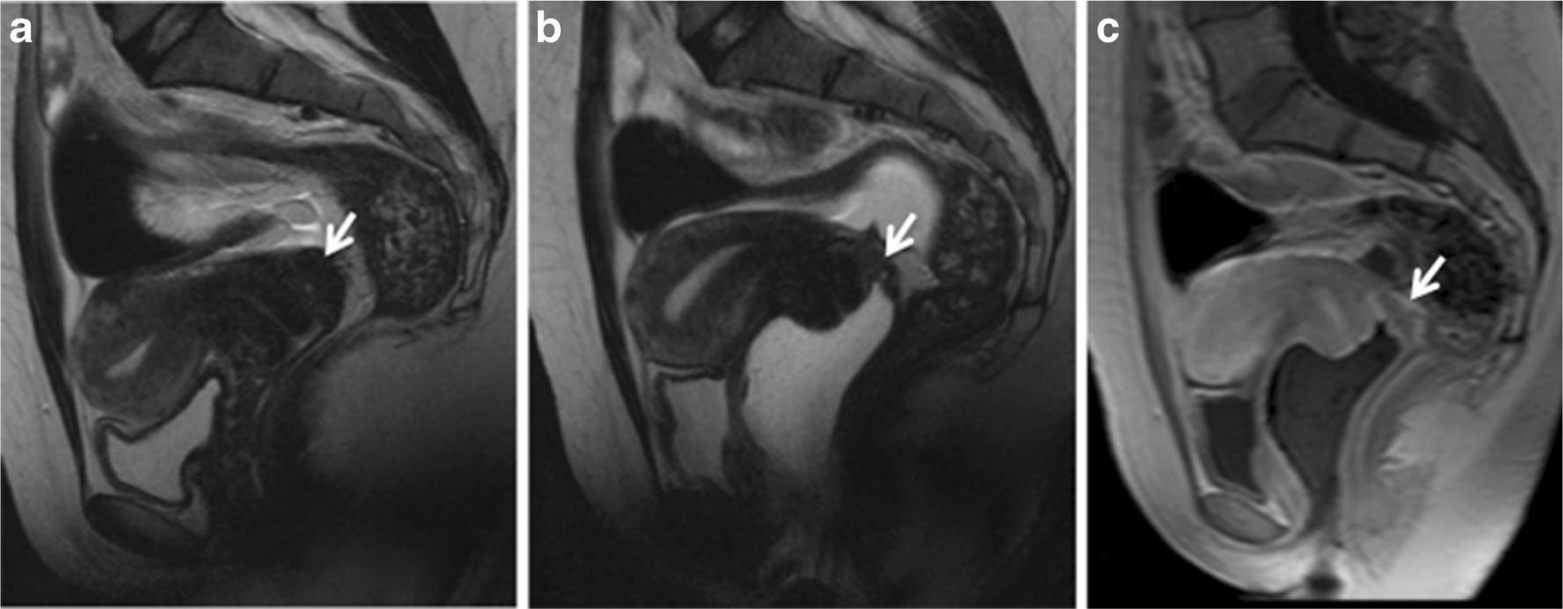
Sagittal 2D MR images performed at 1.5 Tesla demonstrating the use of sonographic gel to opacify and distend the vagina. (**a**) Sagittal 2D T2-weighted image demonstrating an endometriotic plaque involving the posterior vaginal fornix (white arrow). Following distension of the vagina with sonographic gel, the plaque is better delineated on both T2-weighted (**b**) and fat-suppressed T1-weighted (**c**) sequences (white arrows) (reprinted with permission - Bazot M. Ed. Lavoisier-Paris)

Three studies reported data for the evaluation of different DPE locations [14, 61, 62]. Firstly, Onbas et al. suggested that dynamic MRI could be useful to depict abdominal wall endometriosis [61]. Secondly, Scardapane et al. underlined that the combination of MR colonography and 3D-T1W MRI allows easier recognition of colorectal endometriosis and higher inter-observer agreement (LE3) [62]. Finally, Bazot et al. suggested the absence of benefit of intravenous gadolinium for the diagnosis of rectosigmoid colon, vaginal and bladder endometriosis, whatever the level of expertise of readers (LE3) (Fig. 5) [14].

**Fig. 6:**
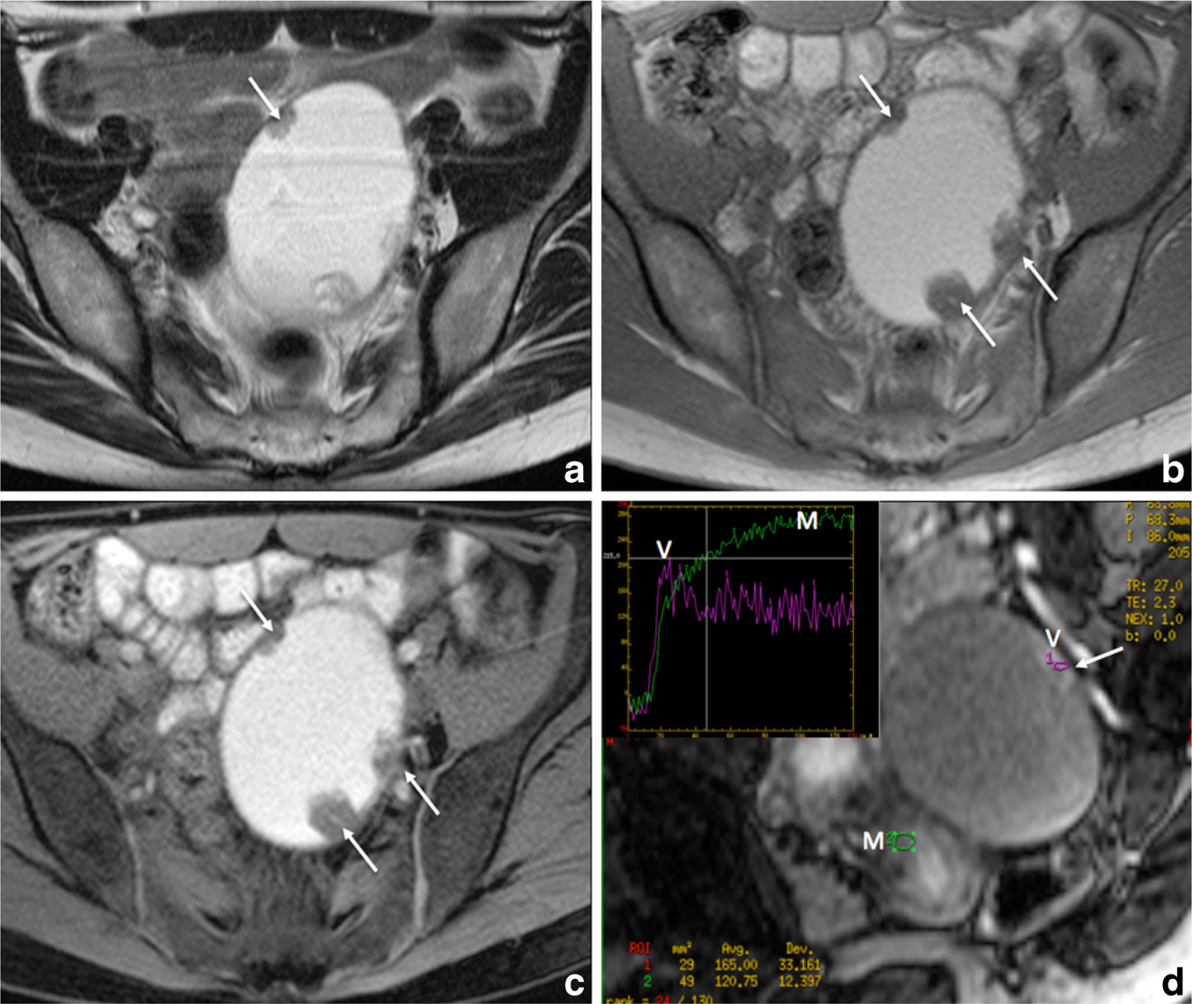
Axial 2D MR images performed at 1.5 Tesla demonstrating the use of gadolinium in the diagnosis of indeterminate adnexal mass related to endometrial cyst complicated with clear cell carcinoma. (**a**) Axial 2D T2-weighted image demonstrates a large unilocular cyst containing papillary projections and/or solid portion (arrows). Axial without (**b**) and with (**c**) fat-suppressed T1-weighted sequences display high signal content related to endometriotic fluid. Axial oblique dynamic contrast enhanced MR images (**d**) display location of region of interest (ROI) within external myometrium (M) and vegetation (V) and the initial increase in the signal intensity of solid tissue (arrow) that is steeper than that of myometrium (M), corresponding to a curve type 3 (V) highly suggestive of carcinoma confirmed at histopathological examination

These variable results contrast with the usefulness of gadolinium when dealing with endometriotic adnexal masses. Using conventional T2W and T1W sequences, MRI has only moderate accuracy in distinction of endometrial cysts from other haemorrhagic adnexal lesions [63, 64]. The use of gadolinium may help to distinguish an endometrioma from a luteal ovarian cyst or tubo-ovarian abscess displaying intense wall enhancement [65]. Moreover, gadolinium enhancement is crucial for depicting strongly enhancing mural nodules if atypical features suggest potential malignancy on either ultrasound (US) or T2W (LE4) (Fig. 6) [66, 67]. Finally, endometriosis and pelvic inflammatory disease are two conditions that can be easily confused, especially in situations where they co-exist. Hence, the presence of a strong wall enhancement within adnexal masses is useful to suggest pelvic inflammatory disease (LE3) [65, 68].

No recommendation can be achieved regarding the use of gadolinium in the evaluation of DPE (Fig. 5).

The use of gadolinium is recommended as an ‘option’ in the evaluation of indeterminate adnexal endometriosis (grade C).

#### Diffusion-weighted MRI (DWI)

No data are available on DWI for the evaluation of DPE. Two recent studies have suggested its potential value in the evaluation of abdominal wall and sacral nerve root abnormalities endometriosis (LE4) [37, 69, 70].

A role for DWI has also been suggested in differentiation of endometriomas from haemorrhagic cysts with significantly lower ADC values in endometriomas when compared with haemorrhagic ovarian cysts at all b values [71].

No recommendation can be achieved for the use of DWI in the evaluation of DPE.

#### Susceptibility-weighted MRI (SWI)

Two recent studies using SWI suggest that the presence of signal voids reflecting acute to chronic haemorrhage are very sensitive in the diagnosis of extra-ovarian endometriosis, especially abdominal wall endometriosis (LE4) [37, 72].

Endometriotic cysts contain blood degradation products secondary to recurrent cyclic bleeding from ectopic endometrial tissue giving rise to punctate or curvilinear signal voids along the cyst wall on SWI [57, 73]. However, a pitfall of imaging with SWI is susceptibility artefact caused by intestinal gas, particularly at 3.0T.

No recommendation can be proposed for the use of susceptibility-weighted MR imaging in the evaluation of deep endometriosis.

#### Half-Fourier acquisition single shot turbo spin echo

Half-Fourier acquisition single shot turbo-spin-echo (SSFSE, HASTE) enables multiphase and multislice image acquisition producing kinematic images for the evaluation of pelvic adhesions [74].

HASTE imaging is used to evaluate uterine function by assessing uterine peristalsis, identifiable as rhythmic and subtle wave-like endometrial and subendometrial myometrium movements [75–77]. During the peri-ovulatory phase, uterine peristalsis is significantly reduced in subjects with endometriosis when compared to normal controls that may be due to increased, sustained contractions in endometriosis patients (LE4) [36, 78]. This abnormal uterine peristalsis in endometriosis patients could interfere with fertility [79].

Half-Fourier acquisition single shot turbo spin echo is recommended for the evaluation of uterine peristalsis (grade C).

#### Reporting criteria

A consensus exists in the ESUR group and in the literature about the criteria used in the diagnosis of endometrial cysts [57] and different locations of DPE [15, 33] (Appendix 1).

## Conclusion

These recommendations argue that:

1. MRI should be considered as a second-line technique examination after US in the evaluation of pelvic endometriosis
2. MRI is recommended before surgery for optimal preoperative staging
3. Some requirements for the acquisition of MR images have to be observed to provide optimal studies.

Future research for the evaluation of endometriosis using MRI:

1. Patients with clinical suspicion and negative US
2. Intra-individual comparison between 1.5T and 3.0T
3. Management of medical treatment
4. Diagnostic performance without and with bowel preparation to evaluate DPE
5. Evaluation of the clinical impact of MRI as a pre-operative test.

## Electronic supplementary material

Below is the link to the electronic supplementary material.

ESM 1 (DOCX 133 kb)

## Abbreviations

2D: Two-dimensional
3D: Three-dimensional
DPE: Deep pelvic endometriosis
DWI: Diffusion-weighted imaging
ESUR: European Society of Urogenital Radiology
FPI-ESUR: Female Pelvic Imaging working group of the European Society of Urogenital Radiology
GPP: Good practice point
HASTE: Half-Fourier acquisition single shot turbo-spin-echo
IM: Imtramuscular
IV: Intravenous
LE: Level of evidence
MRI: Magnetic resonance imaging
OCEBM: Oxford Centre for Evidence Based Medicine
SC: Subcutaneous
SNR: Signal-to-noise ratio
SWI: Susceptibility-weighted imaging
T: Tesla
T1W: T1-weighted
T2W: T2-weighted
TVS: Transvaginal sonography
US: Ultrasound
