# Supplementary material for: European society of urogenital radiology (ESUR) guidelines: MR imaging of pelvic endometriosis
Source: Eur Radiol. 2016 Dec 5;27(7):2765–75. doi: 10.1007/s00330-016-4673-z (PMC5486785; doi:10.1007/s00330-016-4673-z)
Supplement: Supplementary file 1 — (DOCX 133 kb) [file 330_2016_4673_MOESM1_ESM.docx]

**Appendix 1: Radiological Diagnostic Criteria in Endometriosis**

**Reporting criteria**

A consensus exists in the ESUR group and in the literature about the criteria used in the diagnosis of endometrial cysts [59] and different locations of deep pelvic endometriosis [15-35].

**Endometrial cysts**

MRI criteria used to describe an endometrioma and the features that suggest malignant transformation are reported in Tables 4a and 4b.

**Table 4a:** MRI Criteria – Diagnosis of endometrial cysts. This table shows the MRI criteria used to make a diagnosis of an endometrial cyst and the number of experts who were in agreement with each criterion (out of a total of 8).

| Endometrial Cyst | Agreement /8 |
| --- | --- |
| High signal intensity ≥ subcutaneous fat on T1W | 8 |
| High signal intensity not decreased on T1W with fat-sat | 8 |
| Haemorrhagic products often get brighter on T1FS imaging than on standard T1W series while protein/mucin does not tend to do this | 8 |
| T2W shading | 8 |
| Bilaterality | 7 |
| Multiplicity | 8 |
| Shape | 4 |
| Normal ovarian tissue | 8 |
| Position of endometriomas within ovary | 7 |

**Table 4b:** MRI Criteria – Features suggesting malignant transformation within an endometrial cyst. This table shows the MRI criteria used to make a diagnosis of malignant transformation and the number of experts who were in agreement with each criterion (out of a total of 8).

| Endometrial cyst suspected of malignancy | Agreement /8 |
| --- | --- |
| Solid nodule in a cyst | 8 |
| Intermediate T2W signal of solid nodule | 8 |
| Septal thickness >3mm | 4 |
| Ascites | 4 |
| Peritoneal metastases | 8 |
| Solid mass | 5 |
| No context of pregnancy | 8 |

**Deep Pelvic Endometriosis (DPE)**

All members of the ESUR group agree that the diagnosis of DPE is based on the joint presence of signal and morphologic abnormalities [35]:

- Tissue areas corresponding to fibrosis, with a signal close to that of pelvic muscle on T1W and T2W images
- Hyperintense foci on T1W and/or fat-suppression T1W MR images, corresponding to haemorrhagic foci
- Small hyperintense cavities on T2W images.

Morphologic abnormalities with regular or irregular stellate margins are evaluated at each site of posterior or anterior DPE. The abnormalities vary according to the anatomical location as detailed in Table 5.

Rare locations of endometriotic involvement should be evaluated if there is a high clinical suspicion of involvement.

**Adhesions**

On MRI, adhesions are identified as spiculated low signal intensity strands of variable thickness extending between the organs on both T1W and T2W images. In addition, the indirect signs of adhesions have to be evaluated. The most frequent features are represented by distortion of normal anatomy including elevation of the posterior vaginal fornix, posterior and lateral displacement of the uterus, ovaries, or both, loss of fat planes between the structures without a clear interface, hydrosalpinx, angulation of bowel loops, transition points in bowel diameter, and loculated fluid collections [82].

**Peritoneal endometriosis**

Several MR studies have reported data on the value of MRI for the diagnosis of peritoneal endometriosis [6, 16, 44, 46, 57, 62, 83,84]. All these papers are in accordance with European Society of Human Reproduction and Embryology (ESHRE) recommendations suggesting that pelvic MRI is not recommended for the diagnosis of peritoneal endometriotic locations due to the low diagnostic sensitivity of MRI in this setting [2].

**Table 5:** MRI Criteria – Diagnosis of deep pelvic endometriosis by location [6]

| Location | Definitions | Agreement /8 |
| --- | --- | --- |
| Torus uterinus | Presence of a mass or thickening in the upper mid-portion of the posterior cervix. | 8 |
| Uterosacral ligament (USL) | Involvement by endometriosis present when ligament bears a nodule or shows fibrotic thickening compared to the contralateral USL, with regular or irregular margins.  When bilateral involvement is associated with the torus uterinus it is termed an arciform abnormality | 8 |
| Vagina | Obliteration of the hypointense signal of the posterior vaginal wall/ posterior vaginal fornix on T2W images, with thickening or a mass (containing or not containing foci of high T2W SI) behind the posterior wall of the cervix | 8 |
| Rectovaginal septum | Nodule or mass passing through the lower border of the posterior lip of the cervix (under the peritoneum) | 8 |
| Cervix | Thickening of the posterior lip on T2W images, containing or not containing foci of high T2W SI | 8 |
| Rectosigmoid | Disappearance of fat tissue plane lying between uterus and rectum/sigmoid colon, replacement by a tissue mass which form an obtuse angle with the wall of the rectosigmoid | 4  4/8 members felt this alone was not sufficient |
|  | Disappearance of the hypointense signal of the anterior wall of the rectum/sigmoid colon on T2W images | 7 |
|  | Contrast enhancement on T1W images | 4 |
| External Adenomyosis | Nodule / plaque infiltrates into the myometrium from the serosal surface in continuity with DPE | 8 |
| Pouch of Douglas / rectouterine space | Partial or complete obliteration with presence or absence of suspended or lateralised fluid collection | 8 |
| Parametrium | Low-signal-intensity area on T2W MRI, with or without tiny high T2W SI spots in the paracervical or paravaginal region | 8 |
|  | Unilateral (or bilateral) ureteral dilatation | 8 |
| Bladder | Nodule or mass usually located at level of vesicouterine pouch, forming an obtuse angle with the bladder wall | 8 |
|  | Extension through bladder wall involving muscular layer (obliteration of hypointense signal of wall on T2W), or protruding into lumen with invasion of the mucosal layer | 8 |
| Round ligament | Involvement by endometriosis present when ligament shows fibrotic thickening (generally > 1cm) compared to the contralateral round ligament, with regular or irregular margins and occasionally a nodular appearance. | 7 |

DPE – Deep pelvic endometriosis; SI – signal intensity; T1W – T1-weighted; T2W – T2-weighted
